# Data of worldwide observational studies of adults with accelerometry-measured physical activity and sedentary behavior

**DOI:** 10.1016/j.dib.2023.109020

**Published:** 2023-02-28

**Authors:** Kelly R. Evenson, Elissa Scherer, Carmen C. Cuthbertson, Kennedy M. Peter-Marske, Gabriel J. Madson, Stephanie Eckman

**Affiliations:** a Department of Epidemiology, Gillings School of Global Public Health, University of North Carolina – Chapel Hill, Chapel Hill, North Carolina, United States; b RTI International, Research Triangle Park, North Carolina, United States; c Department of Health Education and Promotion, East Carolina University, Greenville, North Carolina, United States

**Keywords:** Accelerometer, Cohort, Epidemiology, Physical behavior, Sitting, Surveillance, Wearable device, NA, not applicable, NI, not indicated

## Abstract

A compendium of observational studies of adults that collected accelerometry to assess physical activity and sedentary behavior (i.e., physical behaviors) could facilitate cross-study comparisons, meta-analyses, and future research collaborations. Therefore, we performed a systematic search to identify observational studies, including surveillance systems, that collected accelerometry-measured physical activity and sedentary behavior among adults. We performed a search using PubMed, Web of Science, and SPORTDiscus for studies published on or before June 1, 2021. After screening 5686 abstracts and 1027 full text articles, we included 155 unique studies that collected accelerometry on at least 500 adults 18 years or older. Most studies used one accelerometer (n=146), although eight studies used two accelerometers and one study used four accelerometers. The country of data collection, age range, and accelerometer characteristics were abstracted and checked by a second reviewer. These datasets summarizing relevant observational studies of adults can be a resource to researchers seeking to identify data sources for accelerometer-measured physical activity and sedentary behavior from around the world.

**Specifications Table**

**Table:** 

Subject	Epidemiology

Specific subject area	Observational studies of adults that collected accelerometry-measured physical activity and sedentary behavior
Type of data	Table
How the data were acquired	Data were acquired through systematic searches of PubMed, Web of Science, and SPORTDiscus databases, and were reviewed and abstracted by at least two coders.
Data format	Raw
Description of data collection	Included surveillance, cross-sectional, and cohort studies were published in English and collected accelerometry to measure physical activity and sedentary behavior on at least 500 adults. Excluded studies included those of hospitalized or institutionalized populations, those that used pedometers, and those that were experimental (e.g., randomized controlled trial or intervention).
Data source location	Studies from around the world identified through three databases: PubMed, Web of Science, and SPORTDiscus
Data accessibility	Repository: University of North Carolina – Chapel Hill Dataverse Cohort level dataset [1]: https://doi.org/10.15139/S3/ZYVHUO Accelerometer level dataset [2]: https://doi.org/10.15139/S3/8GPGJZ
Related research article	[3] Evenson KR, Scherer E, Peter KM, Cuthbertson CC, and Eckman S. Historical development of accelerometry measures and methods for physical activity and sedentary behavior research worldwide: A scoping review of observational studies of adults. PLOS One. 2022 Nov 21;17(11): e0276890.

## Value of the Data

• The datasets can serve as a compendium of observational studies that measured physical activity and sedentary behavior using accelerometry among adults at least 18 years of age.
• The datasets can be used to facilitate future data harmonization analyses, systematic reviews, and meta-analyses of correlates and determinants of physical activity and sedentary behavior, as well as associations of accelerometry metrics (e.g, time in moderate-to-vigorous physical activity, steps per day) with health outcomes.
• The datasets could be expanded to include health outcomes and data availability across studies.
• The datasets include the country location(s) of participants to facilitate cross-country comparisons.
• The ethical and governance issues to accessing and using each data source is not included in the dataset and would need to be further investigated by the potential user.
• The datasets can provide a historic perspective on the field over the past 22 years (i.e., the time since accelerometry measures were integrated into epidemiologic studies).

## 1 Objective

Accelerometers are device-based sensors that can be used to measure physical activity and sedentary behavior. These devices are often integrated into surveillance and epidemiologic studies of health. With the rise in the use of accelerometry, a comprehensive review on key aspects of the devices and processing decision rules would aid the field. Therefore, we conducted a scoping review of the literature to identify studies with accelerometry measured physical activity or sedentary behavior on at least 500 adults [3]. This article describes the cohort and accelerometer level datasets and provides data dictionaries for both to aid users of these resources. The research supports the call from several groups for a more complete reporting of accelerometer methodology to enhance future harmonization [4,5].

## 2 Data Description

There are two datasets associated with the project: a cohort level and an accelerometer level dataset. The cohort level dataset contains 155 observational studies that collected accelerometry on adult participants identified from the systematic searches. Each row of data contains a different study, with an ID variable, study name, description of study (e.g., distributed data, study design, waves of data collection, study years, start year), description of the sample (e.g., country, gender), and number of accelerometers used. Table 1 provides more detail on each variable in the cohort level or study level dataset.

**Table 1:** Variables and description of the cohort level dataset [1].

Variable (Column Header Name)	Description
Cohort ID (IDCOHORT)	An unique identification number is assigned for each study; Surveillance studies with multiple waves received unique study ID numbers; cohort studies with multiple waves received one unique ID number.
PubMed Identifier (PMID)	Main published study from PubMed that we used to abstract information; note that for some studies, other articles were also used, as well as information obtained by contacting the study directly.
Study name (NAME)	Name of study. When not specified, for most studies we named it using the format <Unnamed-Country-Cohort-Year>. Specifically for distributed data studies, the format was <Unnamed-Distributed-Company name-Cohort-Year>.
Distributed (DISTRIBUTED)	“YES” indicates that the participant provided data indirectly through wearing an activity tracker and agreeing to the terms of use.
Study years (YEARS)	Years of accelerometry data collection; when multiple waves were collected, only the first study wave is indicated.
Start year (STARTYEAR)	Year that accelerometry data collection began.
Country (COUNTRY)	Country the participants lived in.
Gender (GENDER)	Gender breakdown as either male, female, or male and female as reported by the studies.
Sample size (SAMPLESIZE)	Analytic sample size provided in the papers reviewed. Sometimes the numbers were in conflict across papers, so we chose the most reasonable one. This does not represent the starting sample size of the study.
Logbook (LOGBOOK)	Whether a logbook was used in the study, indicated by yes, no, or NI (not indicated).
Population-based study (POPBASED)	Whether or not the study was population-based considering only the paper(s) we reviewed.
Sample weights (WEIGHTS)	Whether or not sample weights were used in the paper(s) we reviewed.
Number of accelerometers (ACCNUM)	Number of accelerometers that the study used in the data collection period.

Most studies used one accelerometer (n=146), although eight studies used two accelerometers and one study used four accelerometers. Therefore, we developed a separate accelerometer level dataset (n=166). Each row of data contains a different study*accelerometer, with an ID variable, study name, description of the accelerometer (e.g., brand, model), and features of the study protocol. Table 2 provides more detail on each variable in the accelerometer level dataset.

**Table 2:** Variables and description of the accelerometer level dataset [2].

Variable (Column Header Name)	Description
Accelerometer ID (IDACC)	An unique identification number is assigned for each study by accelerometer. Each row of data represents a single accelerometer. For example, if a study used two accelerometers, then it would be represented in the dataset with two rows. ID's with a number after the decimal point indicate that they used more than one accelerometer.
Cohort ID (IDCOHORT)	An unique identification number is assigned for each study; Surveillance studies with multiple waves received unique study ID numbers; cohort studies with multiple waves received one unique ID number.
Study name (NAME)	Name of study. When not specified, for most studies we named it using the format <Unnamed-Country-Cohort-Year>. Specifically for distributed data studies, the format was <Unnamed-Distributed-Company name-Cohort-Year>.
Brand (BRAND)	Brand name of the accelerometer.
Model (MODEL)	Model name of the accelerometer; NI is not indicated.
Epoch (EPOCH)	Epoch length used for data collection; NI is not indicated and in some cases the accelerometer did not have this setting.
Sampling frequency (SAMPLEFREQ)	Sampling frequency of the accelerometer; NI is not indicated and in some cases the accelerometer did not have this setting.
Location (LOCATION)	Location of accelerometer wear; NI is not indicated.
Side (SIDE)	Side of accelerometer wear; NI is not indicated.
Attachment (ATTACH)	Attachment method for the accelerometer; NI is not indicated.
Distribution method (DIST)	Distribution method of the accelerometer; NI is not indicated; NA is not applicable and applies to the distributed data studies.
Return method (RETURN)	Return method of the accelerometer; NI is not indicated; NA is not applicable and applies to the distributed data studies.
Days of collection (DAYS)	Number of days of accelerometer data collection from the participants; NI is not indicated.
Weekend days (WEEKEND)	Weekend days required as specified in the paper abstracted; N is no; W indicated two work days were required.
Wear protocol (WEAR)	Accelerometer wear protocol for wake only or a 24-hour period; NI is not indicated.

## 3 Experimental Design, Materials and Methods

Three databases (PubMed, Web of Science, SPORTDiscus) were searched on June 1, 2021, with the search strategy describe elsewhere (see Supplement 2 in Evenson et al. [3]). After removing duplicate citations, two authors independently screened all titles and abstracts before subsequently reviewing full-text articles for eligibility. Both title/abstract and full-text screening were conducted using Covidence software (Veritas Health Innovation, Melbourne, Australia). Discrepancies between authors were resolved by consensus.

In our search strategy we included observational studies, including surveillance studies, with analytical sample sizes of at least 500 community-dwelling adults 18 years and older. The participant must have worn an accelerometer for the purposes of collecting physical activity, sedentary behavior, or both. We included only full-length, peer-reviewed papers published in English. If there was more than one publication identifying a single study that met the inclusion criteria, then we included only one publication to represent the study, using the study that provided the most information we were abstracting. If needed, we sought missing information from other publications captured by the search.

We excluded studies of hospitalized or institutionalized adults, or samples that gave consent by proxy, as well as studies of youth (children or adolescents <18 years of age). We excluded intervention studies (i.e., randomized trials, quasi-experimental trials), unless there was a new consent process that enrolled participants into an observational study. We excluded publications in the grey literature, as well as abstracts, dissertations, and conference proceedings. We excluded studies that used spring-levered pedometers, but included pedometers that used accelerometry. Studies that collected accelerometry, but did not report on physical activity or sedentary behavior, were also excluded. After removing duplicate papers captured from the databases, 5686 titles and abstracts were screened for inclusion. Among those, 1027 full-text studies were further screened and 157 studies were included. Further detail can be found in Evenson et al. [3].

Several caveats to the completeness and accuracy of the databases should be noted. First, we based the information on a primary publication so it is possible that missing abstracted fields may be available in other publications. Second, we attempted to capture data availability, but found this was inconsistently reported. Therefore, the database does not include this information. Despite these caveats, the cohort- and accelerometer-level datasets may be useful to researchers seeking to identify data sources for accelerometer-measured physical activity and sedentary behavior from around the world.

## Ethics Statements

This scoping review did not directly collect information on human subjects.

## CRediT Author Statement

**Kelly R. Evenson**: Conceptualization, Data curation, Writing – Original Draft, Funding acquisition; **Elissa Scherer**: Conceptualization, Data curation, Writing – review and editing, Funding acquisition; **Carmen C. Cuthbertson**: Conceptualization, Data curation, Writing – review and editing; **Kennedy M. Peter-Marske**: Data curation, Writing – review and editing; **Gabriel J. Madson**: Data curation, Writing – review and editing; **Stephanie Eckman**: Conceptualization, Data curation, Writing – review and editing.

## Declaration of Competing Interest

The authors declare that they have no known competing financial interests or personal relationships that could have appeared to influence the work reported in this paper.

The authors declare the following financial interests/personal relationships which may be considered as potential competing interests:

## Data Availability

Cohort level dataset (Original data) (Dataverse). Accelerometer level dataset (Original data) (Dataverse).
